# Bis[μ-(3-acetyl-2-hy­droxy-6-methyl-4*H*-pyran-4-one-κ^3^ *O*:*O*′,*O*′′)]diaqua­tetra­kis­(pyridine-κ*N*)dicopper(II) diperchlorate

**DOI:** 10.1107/S1600536812041608

**Published:** 2012-10-13

**Authors:** Ali Ourari, Wassila Derafa, Sofiane Bouacida, Djouhra Aggoun, Jean-Claude Daran

**Affiliations:** a Laboratoire d’Electrochimie, d’Ingénierie Moléculaire et de Catalyse Redox (LEIMCR), Faculté des Sciences de l’Ingénieur, Université Farhat Abbas, Sétif 19000, Algeria; b Unité de Recherche de Cimie de l’Environnement et Moléculaire Structurale, CHEMS, Université Mentouri-Constantine, 25000, Algeria; c Département Sciences de la Matière, Faculté des Sciences Exactes et Sciences de la Nature et de la Vie, Université Oum El Bouaghi, Algeria; d Laboratoire de Chimie de Coordination, UPR CNRS 8241, 205 route de Narbonne, 31077 Toulouse cedex, France

## Abstract

In the centrosymmetric binuclear cation of the title compound, [Cu(C_8_H_7_O_4_)(H_2_O)(C_5_H_5_N)_2_]_2_(ClO_4_)_2_, the Cu^II^ atoms are bridged by a pair of two dehydro­acetate anions in a bis-/monodentate mode. The distorted octa­hedral N_2_O_4_ coordination sphere of the metal cation is completed by two pyridine N atoms and one O atom of a water mol­ecule. The complex cations and the perchlorate counter anions are arranged in layers parallel to (100). O—H⋯O hydrogen bonds between the coordinating water mol­ecules and the perchlorate anions constitute ribbons parallel to [10-1]. C—H⋯O hydrogen bonds are also observed.

## Related literature

For the synthesis of similar compounds, see: Tan & Kok-Peng Ang (1988 ▶); El-Kubaisi & Ismail (1994 ▶); Danilova *et al.* (2003 ▶); Munde *et al.* (2010 ▶); Ourari *et al.* (2011 ▶). For applications of related compounds, see: Maiti *et al.* (1988 ▶); Mohan *et al.* (1981 ▶); Das & Livingstone (1976 ▶); Ourari *et al.* (2008 ▶, 2012 ▶).
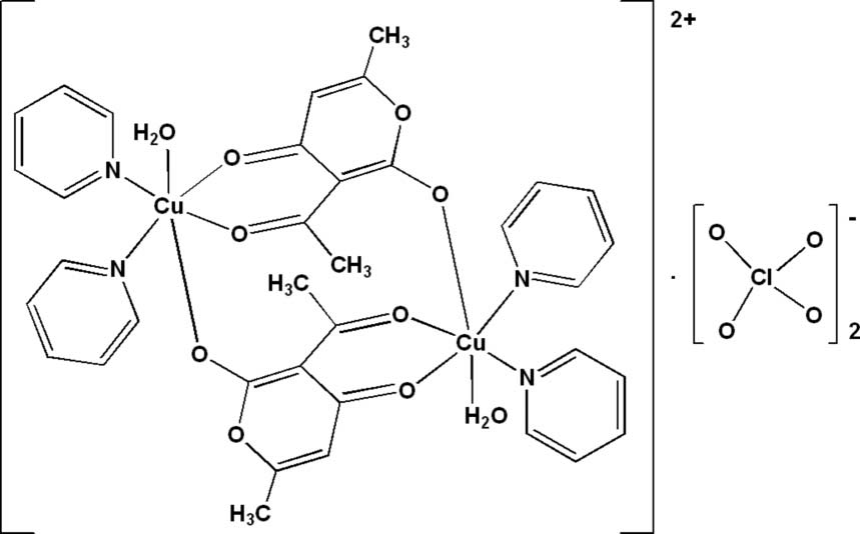


## Experimental

### 

#### Crystal data

[Cu(C_8_H_7_O_4_)(H_2_O)(C_5_H_5_N)_2_]_2_(ClO_4_)_2_
*M* *_r_* = 1012.70
Triclinic, 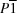

*a* = 9.9371 (4) Å
*b* = 10.3072 (4) Å
*c* = 10.4440 (5) Å
α = 99.624 (4)°
β = 90.540 (3)°
γ = 97.895 (4)°
*V* = 1044.09 (8) Å^3^
*Z* = 1
Mo *K*α radiation
μ = 1.23 mm^−1^
*T* = 180 K
0.44 × 0.34 × 0.13 mm

#### Data collection

Agilent Xcalibur diffractometer
Absorption correction: multi-scan (*CrysAlis PRO*; Agilent, 2011 ▶) *T*_min_ = 0.505, *T*_max_ = 1.000
20280 measured reflections
4692 independent reflections
3889 reflections with *I* > 2σ(*I*)
*R*_int_ = 0.037

#### Refinement

*R*[*F* ^2^ > 2σ(*F* ^2^)] = 0.054
*wR*(*F* ^2^) = 0.140
*S* = 1.12
4692 reflections
288 parameters
H atoms treated by a mixture of independent and constrained refinement
Δρ_max_ = 1.14 e Å^−3^
Δρ_min_ = −0.65 e Å^−3^

### 

Data collection: *CrysAlis PRO* (Agilent, 2011 ▶); cell refinement: *CrysAlis PRO*; data reduction: *CrysAlis PRO*; program(s) used to solve structure: *SIR2002* (Burla *et al.*, 2005 ▶); program(s) used to refine structure: *SHELXL97* (Sheldrick, 2008 ▶); molecular graphics: *ORTEP-3 for Windows* (Farrugia, 1997 ▶) and *DIAMOND* (Brandenburg & Berndt, 2001 ▶); software used to prepare material for publication: *WinGX* (Farrugia, 1999 ▶).

## Supplementary Material

Crystal structure: contains datablock(s) global, I. DOI: 10.1107/S1600536812041608/wm2685sup1.cif

Structure factors: contains datablock(s) I. DOI: 10.1107/S1600536812041608/wm2685Isup2.hkl

Additional supplementary materials:  crystallographic information; 3D view; checkCIF report

## Figures and Tables

**Table 1:** Selected bond lengths (Å)

Cu1—O1	1.922 (3)
Cu1—O2	1.962 (3)
Cu1—N2	2.005 (3)
Cu1—N1	2.006 (3)
Cu1—O1*W*	2.325 (3)
Cu1—O4^i^	2.737 (3)

Symmetry code: (i) 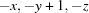.

**Table 2:** Hydrogen-bond geometry (Å, °)

*D*—H⋯*A*	*D*—H	H⋯*A*	*D*⋯*A*	*D*—H⋯*A*
O1*W*—H1*W*⋯O12	0.83 (6)	2.13 (6)	2.934 (9)	165 (6)
O1*W*—H2*W*⋯O11^ii^	0.74 (6)	2.06 (6)	2.772 (9)	164 (6)
C9—H9⋯O13^iii^	0.93	2.56	3.389 (7)	148

Symmetry codes: (ii) 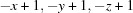; (iii) 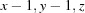.

**Fig. 1.**
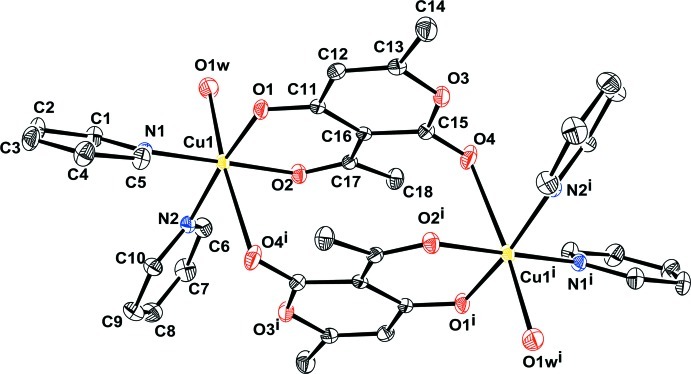
The binuclear complex cation of (I) with the atom-labelling scheme. Displacement ellipsoids are drawn at the 50% probability level. H atoms and perchlorate anions were omitted for clarity. [Symmetry code: (i)-x, -y + 1, -z.]

**Fig. 2.**
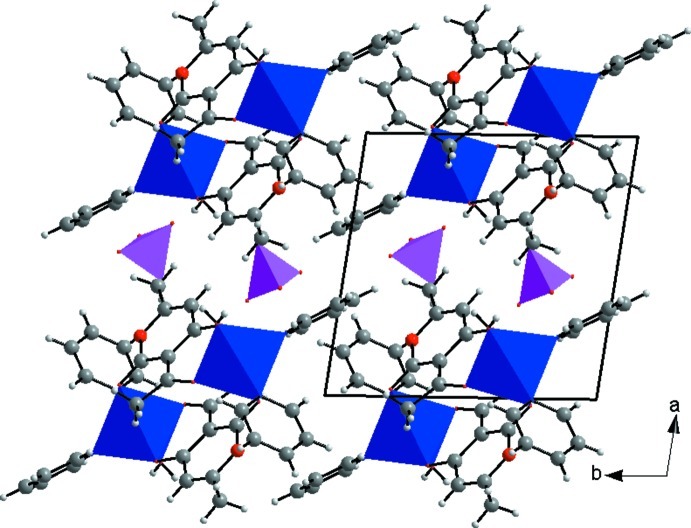
Alternating polyhedra of (I) viewed along [001] showing ClO4 tetrahedra in pink and CuN2O4 octahedra in blue.

**Fig. 3.**
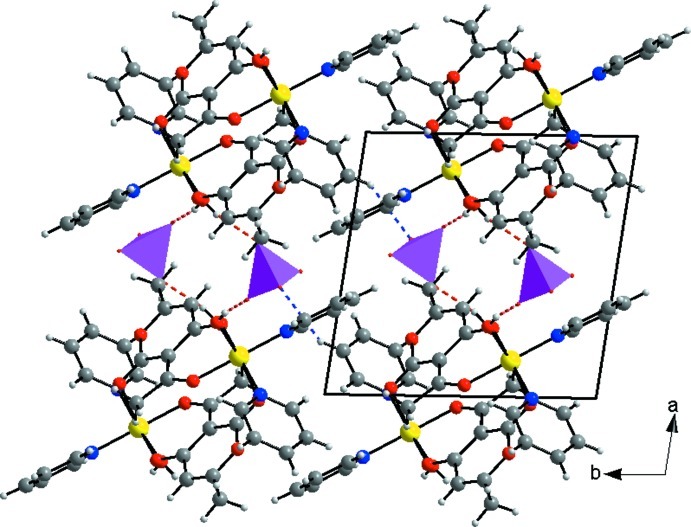
The connection of the components through O—H···O and C—H···O hydrogen bonds (dashed lines).

## supplementary crystallographic information

### Comment

Dehydroacetic acid is used for the synthesis of heterocyclic compounds, some of them with therapeutic activities useful for treatment of human diseases (Das & Livingstone, 1976; Mohan *et al.*, 1981; Maiti *et al.*, 1988). Schiff bases, on the other hand, are widely applied in the synthesis transition metal coordination compounds (Tan & Kok-Peng Ang, 1988; El-Kubaisi & Ismail, 1994; Munde *et al.*, 2010), showing catalytic activities particularly in the oxidation reactions carried out according to the cytochrome P450 model (Ourari *et al.*, 2008, 2011, 2012). Thus, we attempted to synthesize Schiff base half-units in order to use them as starting materials to obtain unsymmetrical tetradentate Schiff base complexes according the Danilova method's (Danilova *et al.*, 2003). Here we describe the formation of a new dinuclear complex, [Cu(C_8_H_7_O_4_)(H_2_O)(C_5_H_5_N)_2_]_2_(ClO_4_)_2_], (I), prepared from dehydroacetic acid, copper perchlorate and pyridine in methanolic solution.

The molecular structure of the complex binuclear and centrosymmetric cation of (I) is illustrated in Fig. 1. The connection mode of the copper cations exhibits dimers, *i.e.* two copper cations are bridged by two dehydroacetate anions in a bis-/monodentate fashion. The asymmetric unit of (I) contains only half of such a dimer. The distorted octahedral coordination sphere around the copper cation is completed by two pyridine ligands and one water molecule. The bond lengths range from 1.922 (3) to 2.325 (3) Å for the Cu—O distances with one more considerably longer bond for Cu—O4 of 2.737 (3) Å; the Cu—N bond lengths are 2.005 (3) and 2.006 (3) Å.

The crystal packing in (I) can be described by alterning layers of cations and tetrahedral perchlorate anions arranged parallel to (100) (Fig. 2). Intermolecular O—H···O hydrogen bonds (Table 2) between the coordinating water molecules and perchlorate anions constitute ribbons parallel to [101]; C—H···O hydrogen bonding interactions eventually links these constituents (Fig. 3).

### Experimental

0.168 g (1 mmol) dehydroacetic acid and 0.373 g (1 mmol) copper bis-perchlorate hexahydrate were dissolved in 20 ml of methanol. To this solution 0.108 g (1 mmol) of 1,2-phenylendiamine was added with an excess of pyridine. The mixture was held under stirring and argon atmosphere for two hours. After that time a precipitate appeared that was recovered by filtration. The solid was washed several times with methanol before it was dried under vacuum (yield 64%). From the resulting filtrate crystals were obtained by slow evaporation.

### Refinement

The H atoms were localized on Fourier maps but introduced in calculated positions and treated as riding on their parent C atom with C—H = 0.96 Å (methyl) or 0.93 Å (aromatic) and with *U*_iso_(H) = 1.2*U*_eq_(C) or *U*_iso_(H) = 1.5*U*_eq_(methyl). H1W and H2W protons of the water molecule were located in a difference Fourier map and were refined isotropically with *U*_iso_(H) = 1.5Ueq(O).

### Crystal data

**Table:**

[Cu(C_8_H_7_O_4_)(H_2_O)(C_5_H_5_N)_2_]_2_(ClO_4_)_2_	*Z* = 1
*M**_r_* = 1012.70	*F*(000) = 518
Triclinic, *P*1	*D*_x_ = 1.611 Mg m^−^^3^
*a* = 9.9371 (4) Å	Mo *K*α radiation, λ = 0.71073 Å
*b* = 10.3072 (4) Å	Cell parameters from 12265 reflections
*c* = 10.4440 (5) Å	θ = 2.6–28.3°
α = 99.624 (4)°	µ = 1.23 mm^−^^1^
β = 90.540 (3)°	*T* = 180 K
γ = 97.895 (4)°	Fragment, dark blue
*V* = 1044.09 (8) Å^3^	0.44 × 0.34 × 0.13 mm

### Data collection

**Table:**

Agilent Xcalibur diffractometer	4692 independent reflections
Radiation source: fine-focus sealed tube	3889 reflections with *I* > 2σ(*I*)
Graphite monochromator	*R*_int_ = 0.037
Detector resolution: 8.2632 pixels mm^-1^	θ_max_ = 28.2°, θ_min_ = 2.7°
ω scans	*h* = −13→11
Absorption correction: multi-scan (*CrysAlis PRO*; Agilent, 2011)	*k* = −13→13
*T*_min_ = 0.505, *T*_max_ = 1.000	*l* = −13→13
20280 measured reflections	

### Refinement

**Table:**

Refinement on *F*^2^	Primary atom site location: structure-invariant direct methods
Least-squares matrix: full	Secondary atom site location: difference Fourier map
*R*[*F*^2^ > 2σ(*F*^2^)] = 0.054	Hydrogen site location: inferred from neighbouring sites
*wR*(*F*^2^) = 0.140	H atoms treated by a mixture of independent and constrained refinement
*S* = 1.12	*w* = 1/[σ^2^(*F*_o_^2^) + (0.0426*P*)^2^ + 3.6572*P*] where *P* = (*F*_o_^2^ + 2*F*_c_^2^)/3
4692 reflections	(Δ/σ)_max_ < 0.001
288 parameters	Δρ_max_ = 1.14 e Å^−^^3^
0 restraints	Δρ_min_ = −0.65 e Å^−^^3^

### Special details

**Table:**

Geometry. All e.s.d.'s (except the e.s.d. in the dihedral angle between two l.s. planes) are estimated using the full covariance matrix. The cell e.s.d.'s are taken into account individually in the estimation of e.s.d.'s in distances, angles and torsion angles; correlations between e.s.d.'s in cell parameters are only used when they are defined by crystal symmetry. An approximate (isotropic) treatment of cell e.s.d.'s is used for estimating e.s.d.'s involving l.s. planes.
Refinement. Refinement of *F*^2^ against ALL reflections. The weighted *R*-factor *wR* and goodness of fit *S* are based on *F*^2^, conventional *R*-factors *R* are based on *F*, with *F* set to zero for negative *F*^2^. The threshold expression of *F*^2^ > σ(*F*^2^) is used only for calculating *R*-factors(gt) *etc*. and is not relevant to the choice of reflections for refinement. *R*-factors based on *F*^2^ are statistically about twice as large as those based on *F*, and *R*- factors based on ALL data will be even larger.

### Fractional atomic coordinates and isotropic or equivalent isotropic displacement parameters (Å^2^)

**Table:**

	*x*	*y*	*z*	*U*_iso_*/*U*_eq_	
Cu1	0.13889 (5)	0.33620 (4)	0.22133 (5)	0.02379 (14)	
Cl1	0.54880 (11)	0.73089 (10)	0.34098 (11)	0.0366 (3)	
O3	0.2147 (3)	0.7115 (3)	−0.0994 (3)	0.0314 (6)	
O4	0.0272 (3)	0.7603 (3)	−0.0061 (3)	0.0397 (7)	
O2	0.0424 (3)	0.4919 (3)	0.2469 (3)	0.0287 (6)	
O1	0.2610 (3)	0.4210 (3)	0.1090 (3)	0.0294 (6)	
O1W	0.2668 (4)	0.4378 (4)	0.4076 (4)	0.0436 (8)	
H1W	0.322 (6)	0.498 (6)	0.387 (6)	0.052*	
H2W	0.304 (6)	0.415 (6)	0.459 (6)	0.052*	
O14	0.5337 (5)	0.8399 (4)	0.4391 (4)	0.0721 (13)	
N1	0.2368 (3)	0.1771 (3)	0.1824 (3)	0.0240 (6)	
N2	−0.0062 (3)	0.2363 (3)	0.3136 (3)	0.0248 (7)	
O13	0.5953 (5)	0.7736 (5)	0.2227 (4)	0.0706 (12)	
C12	0.3329 (4)	0.5426 (4)	−0.0505 (4)	0.0270 (8)	
H12	0.404	0.4928	−0.0678	0.032*	
C1	0.2806 (4)	0.1181 (4)	0.2765 (4)	0.0293 (8)	
H1	0.2662	0.1531	0.3625	0.035*	
C16	0.1369 (4)	0.5980 (3)	0.0778 (4)	0.0223 (7)	
C15	0.1196 (4)	0.6938 (4)	−0.0055 (4)	0.0267 (8)	
C2	0.3464 (5)	0.0069 (4)	0.2508 (4)	0.0347 (10)	
H2	0.3773	−0.0312	0.3182	0.042*	
C18	−0.0449 (5)	0.6868 (4)	0.2296 (4)	0.0332 (9)	
H18A	−0.1173	0.6794	0.1662	0.05*	
H18B	0.0055	0.7746	0.2409	0.05*	
H18C	−0.0821	0.6706	0.3109	0.05*	
C5	0.2587 (4)	0.1269 (4)	0.0587 (4)	0.0302 (9)	
H5	0.2309	0.1692	−0.0069	0.036*	
C11	0.2416 (4)	0.5159 (3)	0.0508 (4)	0.0230 (7)	
C4	0.3208 (5)	0.0149 (4)	0.0251 (4)	0.0372 (10)	
H4	0.3326	−0.019	−0.0616	0.045*	
C3	0.3650 (5)	−0.0458 (4)	0.1227 (4)	0.0371 (10)	
H3	0.407	−0.1216	0.1024	0.044*	
C10	−0.0591 (4)	0.1101 (4)	0.2648 (4)	0.0293 (8)	
H10	−0.021	0.0671	0.1914	0.035*	
C13	0.3180 (4)	0.6368 (4)	−0.1201 (4)	0.0277 (8)	
C14	0.4058 (5)	0.6759 (5)	−0.2262 (5)	0.0427 (11)	
H14A	0.4826	0.6283	−0.2323	0.064*	
H14B	0.4368	0.7697	−0.2076	0.064*	
H14C	0.3545	0.6549	−0.3071	0.064*	
C17	0.0485 (4)	0.5860 (4)	0.1837 (4)	0.0230 (7)	
C9	−0.1668 (5)	0.0422 (4)	0.3184 (4)	0.0363 (10)	
H9	−0.1997	−0.0455	0.2829	0.044*	
C8	−0.2253 (5)	0.1065 (5)	0.4261 (5)	0.0395 (10)	
H8	−0.3	0.0637	0.4629	0.047*	
C7	−0.1711 (5)	0.2350 (5)	0.4780 (4)	0.0410 (11)	
H7	−0.2075	0.2797	0.5515	0.049*	
O11	0.6447 (9)	0.6599 (10)	0.3795 (6)	0.179 (5)	
O12	0.4201 (7)	0.6613 (8)	0.3069 (6)	0.140 (3)	
C6	−0.0622 (5)	0.2967 (4)	0.4196 (4)	0.0318 (9)	
H6	−0.0261	0.3835	0.455	0.038*	

### Atomic displacement parameters (Å^2^)

**Table:**

	*U*^11^	*U*^22^	*U*^33^	*U*^12^	*U*^13^	*U*^23^
Cu1	0.0241 (3)	0.0198 (2)	0.0295 (2)	0.00679 (17)	0.00515 (18)	0.00681 (17)
Cl1	0.0365 (6)	0.0255 (5)	0.0483 (6)	0.0053 (4)	0.0024 (5)	0.0072 (4)
O3	0.0299 (16)	0.0301 (14)	0.0389 (16)	0.0094 (12)	0.0052 (12)	0.0152 (12)
O4	0.0356 (17)	0.0455 (18)	0.0476 (18)	0.0221 (14)	0.0078 (14)	0.0220 (15)
O2	0.0314 (15)	0.0247 (13)	0.0324 (14)	0.0084 (11)	0.0070 (12)	0.0075 (11)
O1	0.0255 (15)	0.0268 (14)	0.0412 (16)	0.0114 (11)	0.0075 (12)	0.0143 (12)
O1W	0.050 (2)	0.0353 (18)	0.0444 (19)	0.0003 (15)	−0.0157 (16)	0.0085 (15)
O14	0.107 (4)	0.050 (2)	0.054 (2)	0.015 (2)	−0.005 (2)	−0.0095 (18)
N1	0.0224 (16)	0.0226 (15)	0.0284 (16)	0.0053 (12)	0.0029 (13)	0.0068 (12)
N2	0.0257 (17)	0.0219 (15)	0.0277 (16)	0.0055 (13)	0.0012 (13)	0.0044 (12)
O13	0.080 (3)	0.078 (3)	0.057 (2)	0.005 (2)	0.007 (2)	0.028 (2)
C12	0.0186 (19)	0.0291 (19)	0.034 (2)	0.0055 (15)	0.0027 (16)	0.0067 (16)
C1	0.033 (2)	0.030 (2)	0.0273 (19)	0.0101 (17)	0.0069 (16)	0.0080 (15)
C16	0.0181 (18)	0.0178 (16)	0.0297 (18)	−0.0008 (14)	−0.0027 (14)	0.0030 (14)
C15	0.0223 (19)	0.0242 (18)	0.034 (2)	0.0038 (15)	−0.0024 (16)	0.0063 (15)
C2	0.041 (3)	0.032 (2)	0.036 (2)	0.0147 (19)	0.0022 (19)	0.0151 (17)
C18	0.037 (2)	0.032 (2)	0.034 (2)	0.0153 (18)	0.0069 (18)	0.0038 (17)
C5	0.033 (2)	0.031 (2)	0.0293 (19)	0.0107 (17)	0.0014 (17)	0.0066 (16)
C11	0.0191 (18)	0.0190 (16)	0.0306 (19)	0.0011 (14)	−0.0028 (15)	0.0050 (14)
C4	0.044 (3)	0.035 (2)	0.032 (2)	0.016 (2)	−0.0001 (19)	−0.0030 (17)
C3	0.039 (3)	0.030 (2)	0.045 (2)	0.0190 (19)	0.002 (2)	0.0024 (18)
C10	0.029 (2)	0.0258 (19)	0.032 (2)	0.0012 (16)	0.0049 (17)	0.0031 (15)
C13	0.0200 (19)	0.0275 (19)	0.035 (2)	0.0015 (15)	−0.0001 (16)	0.0067 (16)
C14	0.038 (3)	0.045 (3)	0.052 (3)	0.011 (2)	0.015 (2)	0.022 (2)
C17	0.0201 (18)	0.0207 (17)	0.0277 (18)	0.0035 (14)	−0.0028 (14)	0.0020 (14)
C9	0.037 (2)	0.032 (2)	0.038 (2)	−0.0039 (18)	0.0037 (19)	0.0062 (18)
C8	0.032 (2)	0.048 (3)	0.041 (2)	0.000 (2)	0.0091 (19)	0.016 (2)
C7	0.045 (3)	0.045 (3)	0.036 (2)	0.012 (2)	0.019 (2)	0.0074 (19)
O11	0.250 (9)	0.288 (10)	0.075 (4)	0.234 (9)	0.050 (5)	0.081 (5)
O12	0.113 (5)	0.165 (6)	0.098 (4)	−0.085 (5)	0.023 (4)	−0.027 (4)
C6	0.037 (2)	0.029 (2)	0.028 (2)	0.0066 (17)	0.0056 (17)	0.0011 (16)

### Geometric parameters (Å, º)

**Table:**

Cu1—O1	1.922 (3)	C16—C11	1.431 (5)
Cu1—O2	1.962 (3)	C16—C15	1.447 (5)
Cu1—N2	2.005 (3)	C2—C3	1.382 (6)
Cu1—N1	2.006 (3)	C2—H2	0.93
Cu1—O1W	2.325 (3)	C18—C17	1.509 (5)
Cu1—O4^i^	2.737 (3)	C18—H18A	0.96
Cl1—O11	1.374 (5)	C18—H18B	0.96
Cl1—O12	1.390 (6)	C18—H18C	0.96
Cl1—O14	1.414 (4)	C5—C4	1.379 (5)
Cl1—O13	1.439 (4)	C5—H5	0.93
O3—C13	1.363 (5)	C4—C3	1.380 (6)
O3—C15	1.386 (5)	C4—H4	0.93
O4—C15	1.219 (5)	C3—H3	0.93
O2—C17	1.256 (4)	C10—C9	1.373 (6)
O1—C11	1.269 (4)	C10—H10	0.93
O1W—H1W	0.82 (6)	C13—C14	1.491 (6)
O1W—H2W	0.74 (6)	C14—H14A	0.96
N1—C1	1.337 (5)	C14—H14B	0.96
N1—C5	1.340 (5)	C14—H14C	0.96
N2—C6	1.341 (5)	C9—C8	1.383 (6)
N2—C10	1.346 (5)	C9—H9	0.93
C12—C13	1.329 (5)	C8—C7	1.377 (7)
C12—C11	1.437 (5)	C8—H8	0.93
C12—H12	0.93	C7—C6	1.378 (6)
C1—C2	1.385 (5)	C7—H7	0.93
C1—H1	0.93	C6—H6	0.93
C16—C17	1.430 (5)		
			
O1—Cu1—O2	89.43 (12)	C1—C2—H2	120.8
O1—Cu1—N2	171.16 (14)	C17—C18—H18A	109.5
O2—Cu1—N2	90.52 (13)	C17—C18—H18B	109.5
O1—Cu1—N1	88.01 (13)	H18A—C18—H18B	109.5
O2—Cu1—N1	176.25 (14)	C17—C18—H18C	109.5
N2—Cu1—N1	91.58 (14)	H18A—C18—H18C	109.5
O1—Cu1—O1W	92.98 (14)	H18B—C18—H18C	109.5
O2—Cu1—O1W	86.49 (13)	N1—C5—C4	122.4 (4)
N2—Cu1—O1W	95.84 (14)	N1—C5—H5	118.8
N1—Cu1—O1W	96.38 (14)	C4—C5—H5	118.8
O1—Cu1—O4^i^	87.05 (12)	O1—C11—C16	125.5 (4)
O2—Cu1—O4^i^	87.41 (12)	O1—C11—C12	117.0 (3)
N2—Cu1—O4^i^	84.12 (13)	C16—C11—C12	117.6 (3)
N1—Cu1—O4^i^	89.71 (12)	C5—C4—C3	118.7 (4)
O1W—Cu1—O4^i^	173.90 (11)	C5—C4—H4	120.6
O11—Cl1—O12	116.7 (6)	C3—C4—H4	120.6
O11—Cl1—O14	110.3 (4)	C4—C3—C2	119.4 (4)
O12—Cl1—O14	107.6 (4)	C4—C3—H3	120.3
O11—Cl1—O13	106.5 (4)	C2—C3—H3	120.3
O12—Cl1—O13	103.9 (4)	N2—C10—C9	122.9 (4)
O14—Cl1—O13	111.7 (3)	N2—C10—H10	118.5
C13—O3—C15	122.2 (3)	C9—C10—H10	118.5
C17—O2—Cu1	129.4 (2)	C12—C13—O3	121.5 (4)
C11—O1—Cu1	127.4 (2)	C12—C13—C14	127.0 (4)
Cu1—O1W—H1W	107 (4)	O3—C13—C14	111.5 (3)
Cu1—O1W—H2W	135 (5)	C13—C14—H14A	109.5
H1W—O1W—H2W	103 (6)	C13—C14—H14B	109.5
C1—N1—C5	118.5 (3)	H14A—C14—H14B	109.5
C1—N1—Cu1	122.0 (3)	C13—C14—H14C	109.5
C5—N1—Cu1	119.5 (3)	H14A—C14—H14C	109.5
C6—N2—C10	117.7 (4)	H14B—C14—H14C	109.5
C6—N2—Cu1	120.9 (3)	O2—C17—C16	123.2 (3)
C10—N2—Cu1	121.1 (3)	O2—C17—C18	114.3 (3)
C13—C12—C11	121.4 (4)	C16—C17—C18	122.4 (3)
C13—C12—H12	119.3	C10—C9—C8	118.8 (4)
C11—C12—H12	119.3	C10—C9—H9	120.6
N1—C1—C2	122.5 (4)	C8—C9—H9	120.6
N1—C1—H1	118.7	C7—C8—C9	118.8 (4)
C2—C1—H1	118.7	C7—C8—H8	120.6
C17—C16—C11	121.5 (3)	C9—C8—H8	120.6
C17—C16—C15	119.6 (3)	C8—C7—C6	119.2 (4)
C11—C16—C15	118.9 (3)	C8—C7—H7	120.4
O4—C15—O3	114.4 (3)	C6—C7—H7	120.4
O4—C15—C16	127.6 (4)	N2—C6—C7	122.5 (4)
O3—C15—C16	118.0 (3)	N2—C6—H6	118.7
C3—C2—C1	118.4 (4)	C7—C6—H6	118.7
C3—C2—H2	120.8		
			
O1—Cu1—O2—C17	14.4 (3)	Cu1—O1—C11—C16	13.9 (6)
N2—Cu1—O2—C17	−156.7 (3)	Cu1—O1—C11—C12	−166.1 (3)
O1W—Cu1—O2—C17	107.5 (3)	C17—C16—C11—O1	5.2 (6)
O2—Cu1—O1—C11	−19.7 (3)	C15—C16—C11—O1	−174.0 (4)
N1—Cu1—O1—C11	157.4 (3)	C17—C16—C11—C12	−174.8 (3)
O1W—Cu1—O1—C11	−106.1 (3)	C15—C16—C11—C12	6.0 (5)
O1—Cu1—N1—C1	130.8 (3)	C13—C12—C11—O1	177.6 (4)
N2—Cu1—N1—C1	−58.0 (3)	C13—C12—C11—C16	−2.4 (6)
O1W—Cu1—N1—C1	38.0 (3)	N1—C5—C4—C3	−1.6 (7)
O1—Cu1—N1—C5	−49.7 (3)	C5—C4—C3—C2	−0.1 (7)
N2—Cu1—N1—C5	121.5 (3)	C1—C2—C3—C4	1.5 (7)
O1W—Cu1—N1—C5	−142.6 (3)	C6—N2—C10—C9	0.2 (6)
O2—Cu1—N2—C6	−39.9 (3)	Cu1—N2—C10—C9	−174.0 (3)
N1—Cu1—N2—C6	143.3 (3)	C11—C12—C13—O3	−0.7 (6)
O1W—Cu1—N2—C6	46.5 (3)	C11—C12—C13—C14	179.1 (4)
O2—Cu1—N2—C10	134.0 (3)	C15—O3—C13—C12	−0.1 (6)
N1—Cu1—N2—C10	−42.8 (3)	C15—O3—C13—C14	−179.9 (4)
O1W—Cu1—N2—C10	−139.6 (3)	Cu1—O2—C17—C16	−2.4 (5)
C5—N1—C1—C2	−0.5 (6)	Cu1—O2—C17—C18	178.1 (3)
Cu1—N1—C1—C2	179.0 (3)	C11—C16—C17—O2	−11.1 (6)
C13—O3—C15—O4	−174.1 (4)	C15—C16—C17—O2	168.1 (4)
C13—O3—C15—C16	3.8 (5)	C11—C16—C17—C18	168.4 (4)
C17—C16—C15—O4	−8.3 (6)	C15—C16—C17—C18	−12.4 (5)
C11—C16—C15—O4	170.9 (4)	N2—C10—C9—C8	1.1 (7)
C17—C16—C15—O3	174.0 (3)	C10—C9—C8—C7	−1.9 (7)
C11—C16—C15—O3	−6.7 (5)	C9—C8—C7—C6	1.4 (7)
N1—C1—C2—C3	−1.2 (7)	C10—N2—C6—C7	−0.7 (6)
C1—N1—C5—C4	2.0 (6)	Cu1—N2—C6—C7	173.5 (3)
Cu1—N1—C5—C4	−177.5 (3)	C8—C7—C6—N2	−0.1 (7)

Symmetry code: (i) −*x*, −*y*+1, −*z*.

### Hydrogen-bond geometry (Å, º)

**Table:**

*D*—H···*A*	*D*—H	H···*A*	*D*···*A*	*D*—H···*A*
O1*W*—H1*W*···O12	0.83 (6)	2.13 (6)	2.934 (9)	165 (6)
O1*W*—H2*W*···O11^ii^	0.74 (6)	2.06 (6)	2.772 (9)	164 (6)
C9—H9···O13^iii^	0.93	2.56	3.389 (7)	148

Symmetry codes: (ii) −*x*+1, −*y*+1, −*z*+1; (iii) *x*−1, *y*−1, *z*.
